# Clinical and genetic characteristics of pachydrusen in patients with exudative age-related macular degeneration

**DOI:** 10.1038/s41598-019-48494-6

**Published:** 2019-08-15

**Authors:** Yoshiko Fukuda, Yoichi Sakurada, Seigo Yoneyama, Wataru Kikushima, Atsushi Sugiyama, Mio Matsubara, Naohiko Tanabe, Hiroyuki Iijima

**Affiliations:** Departments of Ophthalmology, Faculty of Medicine, University of Yamanashi, Yamanashi, Japan

**Keywords:** Genetics research, Genetic testing

## Abstract

We investigated the clinical and genetic characteristics of patients with unilateral exudative age-related macular degeneration (AMD), including typical AMD, polypoidal choroidal vasculopathy, and retinal angiomatous proliferation, in whom pachydrusen was seen. Patients with unilateral exudative AMD with at least a 12-month follow-up period were included. According to the fellow eye condition, 327 consecutive patients were classified into 4 groups: Group 0: no drusen (42.8%), Group 1: pachydrusen (12.2%), Group 2: soft drusen (30.3%), Group 3: pseudodrusen with or without soft drusen (14.7%). Development of exudative AMD in the fellow eye was retrospectively studied for a 60-month period and this inter-group comparisons were performed. Genotyping was performed for *ARMS2* A69S and *CFH* I62V. The thickness of the choroid in the fellow eyes increased significantly in Group 1 than in other groups (all P < 1.0 × 10^−7^). The development of exudative AMD in the fellow eye was significantly less frequent in Group 1 than in Groups 2 or 3 (P = 0.022 and 0.0015, respectively). Risk allele frequency of *ARMS2* A69S was significantly lower in Group 1 than in Group 2 and 3 (all P < 1.0 × 10^−4^). Patients with pachydrusen have genetic and clinical characteristics distinct from those of soft drusen and pseudodrusen.

## Introduction

Drusen are accumulations of extracellular material between the retinal pigment epithelium (RPE) and Bruch’s membrane. They have been considered to result from the normal aging process and may represent early signs of age-related macular degeneration (AMD)^1^. Drusen contain a variety of components such as lipids and inflammatory proteins related to the complement system^2,3^. The risk of advanced AMD differs depending on drusen size and types, presence or absence of pigmentary changes in the RPE, and the condition of the fellow eye^4,5^.

Pseudodrusen are characterized by a yellowish interlacing pattern, are clearly seen on red-free or blue-light fundus photography and are found to be subretinal drusenoid deposits using spectral domain optical coherence tomography (SD-OCT)^6,7^. SD-OCT and near-infrared reflectance imaging are the best modalities for detecting pseudodrusen^8^. Several reports investigating pseudodrusen in the fellow eyes of patients with unilateral neovascular AMD have found that pseudodrusen increases the risk of developing advanced AMD to a greater degree than soft drusen^9–11^.

Pachydrusen is a relatively new entity characterized by isolated or scattered yellow-white deposits, the larger ones exceeding 125 µm in diameter, and having a better-defined outer border. They are seen over the posterior pole of the eye, which has a thickened choroid^12^, and choroidal morphology under pachydrusen showed increased Haller’s layer thickness with attenuation of choriocapillaris^13,14^. SD-OCT revealed that pachydrusen are drusenoid deposits^15,16^. Since no histobiochemical studies have investigated eyes with pachydrusen, it remains unknown whether pachydrusen consist of material similar to that seen in soft drusen and whether they pose the risk of developing exudative AMD.

Genetic variants of *ARMS2* A69S(rs10490924) and *CFH* I62V(rs800292) were strongly associated with exudative AMD in the Asians^17^. Moreover, it has been reported that variants of *ARMS2* are associated with various phenotype including lesion size and bilateral involvement^18–20^.

In the present study, we investigated clinical and genetic factors in patients with unilateral exudative AMD with pachydrusen, soft drusen or pseudodrusen in their unaffected fellow eyes.

## Results

Table 1 presents the genetic and clinical characteristics of patients with unilateral exudative AMD classified into 4 groups according to the drusen types in the fellow eye. The concordance rate between the graders was 96.4% (318/330). A total of 94 patients showed scattered drusen in their fellow eye and late phase ICGA images of 94 eyes were evaluated. Patients in Group 1 (pachydrusen group) were significantly younger than those in Group 2 (soft drusen group) and Group 3 (pseudodrusen group) (p = 3.4 × 10^−4^ and 2.4 × 10^−8^, respectively). T allele frequency of A69S of *ARMS2* gene was significantly lower in Group 1 than in Group 2 and Group 3 (p = 2.4 × 10^−8^ and 2.4 × 10^−5^, respectively). Prevalence of polypoidal choroidal vasculopathy (PCV) was significantly higher in Group 1 (85%) than in the other groups.

**Table 1:** Characteristics of patients with unilateral exudative age-related macular degeneration at initial presentation.

	Group 0 No drusen (n = 140)	Group 1 Pachydrusen (n = 40)	Group 2 Soft drusen (n = 99)	Group 3 Pseudodrusen (n = 48)	P-value
Mean age (year)	71.4	71.4	76.4	81.3	
p-value (vs Pachydrusen)	0.88	NA	3.4 × 10^−4^	2.4 × 10^−8^	6.0 × 10^−12^
Male (%)	106 (75.7%)	32 (80%)	77 (77.8%)	24 (50%)	
p-value (vs Pachydrusen)	0.57	NA	0.77	3.6 × 10^−3^	0.0014
Current smoker (%)	21 (15%)	0 (0%)	20 (20.2%)	4 (8.3%)	
p-value (vs Pachydrusen)	9.2 × 10^−3^	NA	2.1 × 10^−3^	0.061	0.0196
Mean foveal retinal thickness (µm)	187.7	180.5	189.9	180.4	
p-value (vs Pachydrusen)	0.51	NA	0.085	0.63	0.31
*ARMS2* A69S T allele frequency	0.55	0.46	0.71	0.77	
p-value (vs Pachydrusen)	0.18	NA	2.4 × 10^−8^	2.4 × 10^−5^	<0.0001
TT	47 (33.6%)	9 (22.5%)	48 (48.5%)	31 (64.6%)	
TG	59 (42.1%)	19 (47.5%)	45 (45.5%)	12 (25.0%)	
GG	34 (24.3%)	12 (30%)	6 (6.1%)	5 (10.4%)	
*CFH* I62V G allele frequency	0.77	0.63	0.78	0.70	
p-value (vs Pachydrusen)	0.011	NA	9.1 × 10^−3^	0.31	0.029
GG	81 (57.9%)	15 (37.5%)	63 (63.6%)	22 (45.8%)	
GA	53 (37.9%)	20 (50%)	28 (28.3%)	23 (47.9%)	
AA	6 (4.3%)	5 (12.5%)	8 (8.1%)	3 (6.3%)	
AMD subtype					<0.0001
PCV	102 (72.9%)	34 (85%)	47 (47.5%)	7 (14.6%)	
Typical AMD	38 (27.1%)	6 (15%)	44 (44.4%)	34 (70.8%)	
RAP	0 (0%)	0 (0%)	8 (8.1%)	7 (14.6%)	

**Figure 1:**
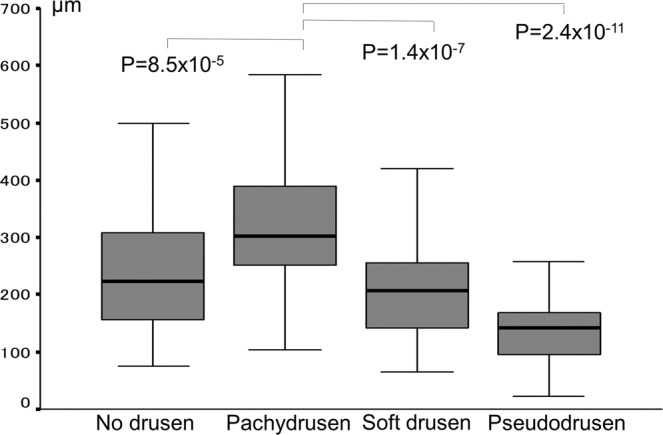
Subfoveal choroidal thickness among the groups.

Mean subfoveal choroidal thickness in Group 1 was 314 µm, which was significantly greater than that in groups 0 (239 µm), 2 (207 µm) and 3 (143 µm) (Fig. 1). Multivariate analysis revealed that mean subfoveal choroidal thickness was still significantly greater in Group1 than in Group 2 and Group 3 (P = 2.2 × 10^−7^ and 3.9 × 10^−9^, respectively) after adjusting age and gender.

**Figure 2:**
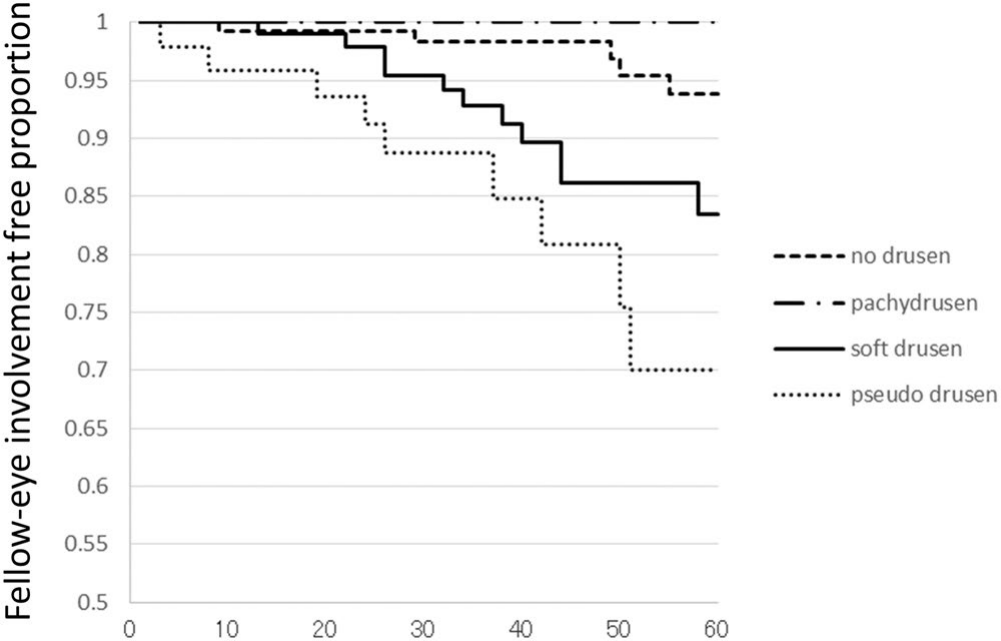
Kaplan-Meier survival estimator showing fellow eye involvement free proportion for 60 months. In Group 0, fellow-eye involvement was seen at 9, 29, 49, 50, and 55 months after first-eye involvement. In Group 2, fellow-eye involvement was seen at 13, 22, 24, 26, 32, 34, 38, 40, 44 (2 eyes), and 58 months after first-eye involvement. In Group 3, fellow-eye involvement was seen at 3, 8, 19, 24, 36, 37, 42, 50, and 51 months after first-eye involvement.

The follow-up periods to involvement of the fellow eye were 43.8 ± 16.3, 47.1 ± 15.5, 42.8 ± 15.7, and 38.1 ± 16.6 months in Groups 0, 1, 2, and 3, respectively, which were not significantly different from one another (p = 0.061, analysis of variance). The number of patients having developed exudative AMD in the fellow eye was 5, 0, 11 and 9 in Groups 0, 1, 2, and 3, respectively. Of 5 fellow eyes developing exudative AMD in Group 0, one eye and four eyes were PCV and typical AMD, respectively. Of 11 fellow eyes developing exudative AMD in Group 2, three eyes, three eyes and five eyes were PCV, typical AMD, and RAP, respectively. Of 9 fellow eyes developing exudative AMD in Group 3, one eye, four eyes, and four eyes were PCV, typical AMD, and RAP, respectively. Kaplan Meier estimator demonstrated a significantly lower risk of developing exudative AMD in the fellow eye for patients in Group 1 compared with those in Groups 2 and 3 (p = 0.022 and 0.0015, respectively, log-rank test). There were no significant differences of the risk between Group 0 and Group 1 (p = 0.21) (Fig. 2).

## Discussion

In the present study, we investigated clinical and genetic characteristics of pachydrusen in comparison with soft drusen and pseudodrusen using patients with unilateral exudative AMD.

Soft drusen are mainly seen in the central macula, but are sometimes scattered around the macula; therefore, it is difficult to differentiate pachydrusen from soft drusen in some cases. Soft drusen contain lipid and many inflammatory materials^21^. It has been reported that hypofluorescent spots on late phase indocyanine green angiography (ICGA) indicate neutral lipid accumulation in Bruch’s membrane and hypofluorescent spots represent “lipid wall” between the basal lamina of RPE and inner collagenous layer of BrM^22^. On the other hand, Tsujikawa *et al*.^23^ reported that punctate hyperfluorescent spots were seen in 38 of 41 eyes (93%) with active central serous chorioretinopathy (CSC) on late phase ICGA. Although they did not demonstrate punctate hyperfluorescent spots on color fundus images with late phase ICGA, it is considered that they may be pachydrusen. We used the late phase ICGA images to differentiate pachydrusen from scattered soft drusen in the present study.

Among 327 patients with unilateral exudative AMD, 190 (58.1%), 122 (37.3%) and 15 (4.6%) showed PCV, typical AMD and retinal angiomatous proliferation respectively. This figure is similar to that in previous reports in the Japanese population^24,25^.

In the present study, Group 1 patients with pachydrusen were significantly younger than those with soft drusen (Group 2) and pseudodrusen (Group 3). Pachydrusen might be early-onset and male-dominant drusenoid deposits while pseudodrusen have been reported to be late-onset and female-dominant^26^.

It has been reported that *ARMS2* A69S and *CFH* I62V are two major genetic variants associated with AMD in the Japanese population and that the risk variant of *ARMS2* A69S is associated with fellow eye involvement in patients with unilateral exudative AMD^27,28^. In the present study, risk (T) allele frequency of *ARMS2* A69S was significantly lower in Group 1 (pachydrusen group) than in Group 2 (soft drusen group) or Group 3 (pseudodrusen group), and the follow-up study revealed that the risk of developing exudative AMD in the fellow eye was significantly lower in Group 1 than in Group 2 or Group 3. Interestingly, risk allele frequency of *ARMS2* A69S was also lower in Group 1 than Group 0 (no drusen group) although it did not reach statistical significance. The results suggest that pachydrusen might not pose a risk of developing exudative AMD but conversely, might have a protective role against developing exudative AMD.

Subfoveal choroidal thickness in the fellow eye was the greatest in Group 1 patients among all 4 groups. It has been reported that choroidal thickness is associated with age, axial length and genetic factors including *ARMS2* and *CFH* variants^29,30^. The patients in Group 1 were the youngest and had the least risk allele frequency of *ARMS2* A69S and *CFH* I62V, which could explain why subfoveal choroidal thickness was the greatest in Group 1 in this study. The axial length was not measured in any of the subjects in the present study.

The frequency of PCV in Group 1 (pachydrusen) was 85%, which was significantly higher than the 47.5% in Group 2 (soft drusen) and 14.3% in Group 3 (pseudodrusen). This finding is consistent with recent reports that drusen subtypes and/or subfoveal choroidal thickness are associated with the type of exudative AMD^12,31^. In the present study, Group1 (pachydrusen group) account for 12.2% among fellow eyes with unilateral exudative AMD and 17.9% among fellow eyes with unilateral PCV. Cheung *et al*.^32^ reported that pachydrusen were seen in 25.5% of exudative AMD on the basis of color fundus photography and Lee *et al*.^33^ reported that pachydrusen were seen in 49.3% of PCV on the basis of multimodal imaging including color fundus photography, SD-OCT, and ICGA. The figure in this study is lower than recent reports^32,33^. Further large studies would be needed to reveal the prevalence of pachydrusen in Asians.

A limitation of the present study is the relatively small sample size of patients with pachydrusen and patients with pseudodrusen. Therefore, the difference in the prevalence of *CFH* I62V variants between Group 1 and Group 3 did not reach statistical significance. In the present study, we did not subdivide Group3 into pseudodrusen with or without soft drusen. Large cohort study will reveal whether they are distinct entities and further studies would be necessary to elucidate the clinical and genetic differences among drusen subtypes.

In summary, pachydrusen have different clinical and genetic characteristics from soft drusen and pseudodrusen. They might be associated with pachychoroid and protection against exudative AMD.

## Method

The medical charts of 330 consecutive patients with unilateral exudative AMD who first visited the Macular Clinic of the Department of Ophthalmology at University of Yamanashi Hospital between August 2011 and August 2017 were retrospectively reviewed. This retrospective study was approved by the institutional review board of the University of Yamanashi and followed the tenets of the Declaration of Helsinki.

**Figure 3:**
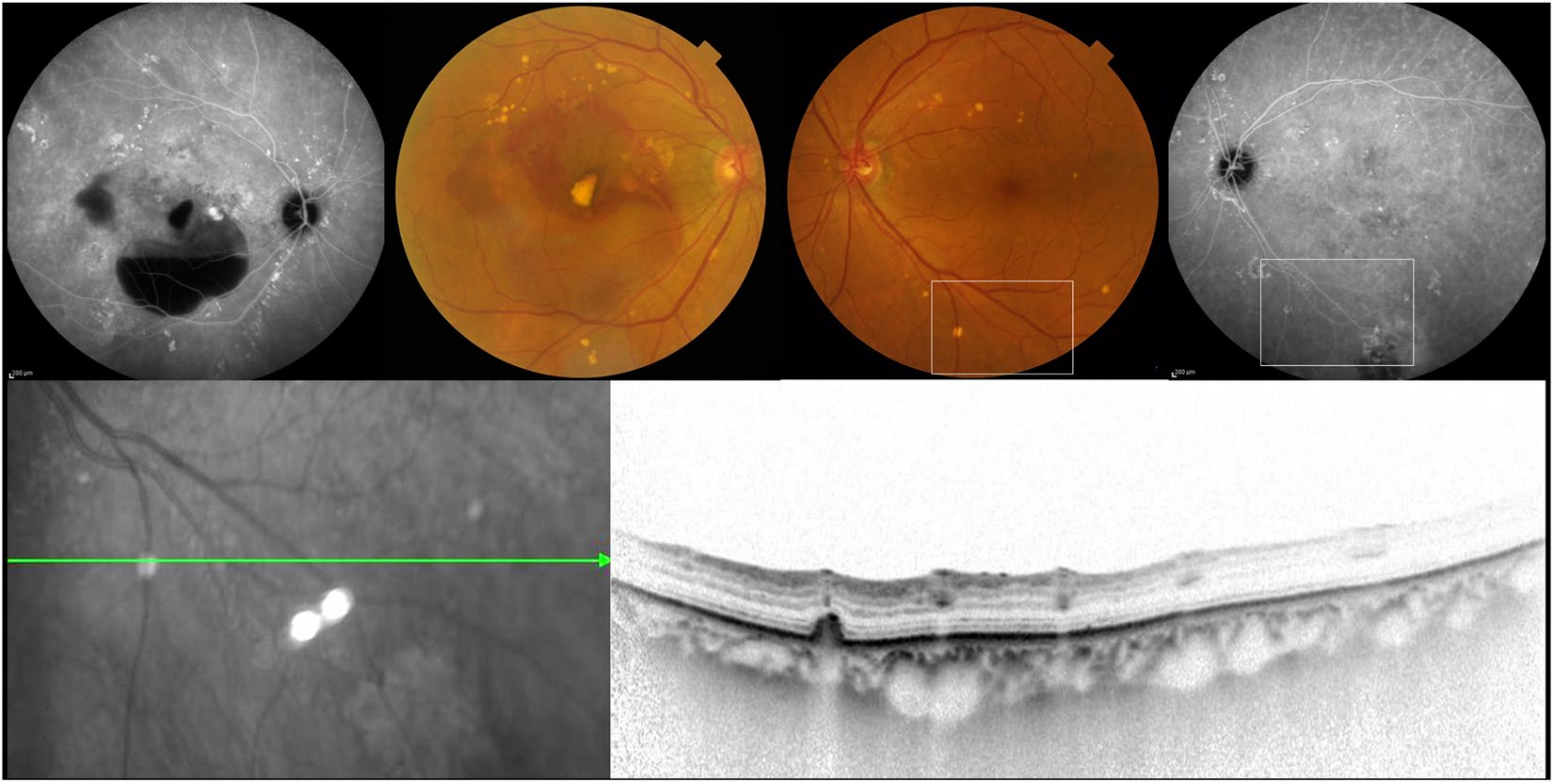
77-year-old female with unilateral polypoidal choroidal vasculopathy and fellow eye with pachydrusen. (**A**,**B**) A color fundus photograph showed subretinal hemorrhage, hemorrhagic pigment epithelial detachment and scattered yellowish drusen in the right eye. Indocyanine green angiography (ICGA) showed polypoidal lesion and multiple hyperfluorescent spots corresponding to yellowish drusen in the right eye. (**C**,**D**) Several yellowish drusen were scattered around the macula on color fundus photography and were found to be hyperfluorescent spots on late phase ICGA. (**E**,**F**) A horizontal optical coherence tomography scan corresponding to white square in Figure (**C**,**D**) showed a drusenoid deposit (pachydrusen) corresponding to a solitary yellowish drusen.

**Figure 4:**
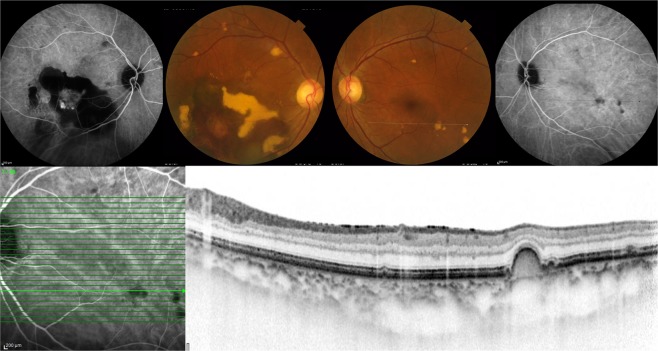
73-year-old male with unilateral polypoidal choroidal vasculopathy and fellow eye with soft drusen. (**A**,**B**) A fundus photograph showed large subretinal hemorrhage in the macular area in the right eye. Indocyanine green angiography (ICGA) showed polypoidal lesion in the right eye. (**C**,**D**) A fundus photograph showed several drusen within the arcade in the left eye. Several drusen showed hypofluorescence on late phase ICGA. (**E**,**F**) Optical coherence tomography demonstrated hypofluorescent spots on ICGA that were RPE bump (soft drusen).

**Figure 5:**
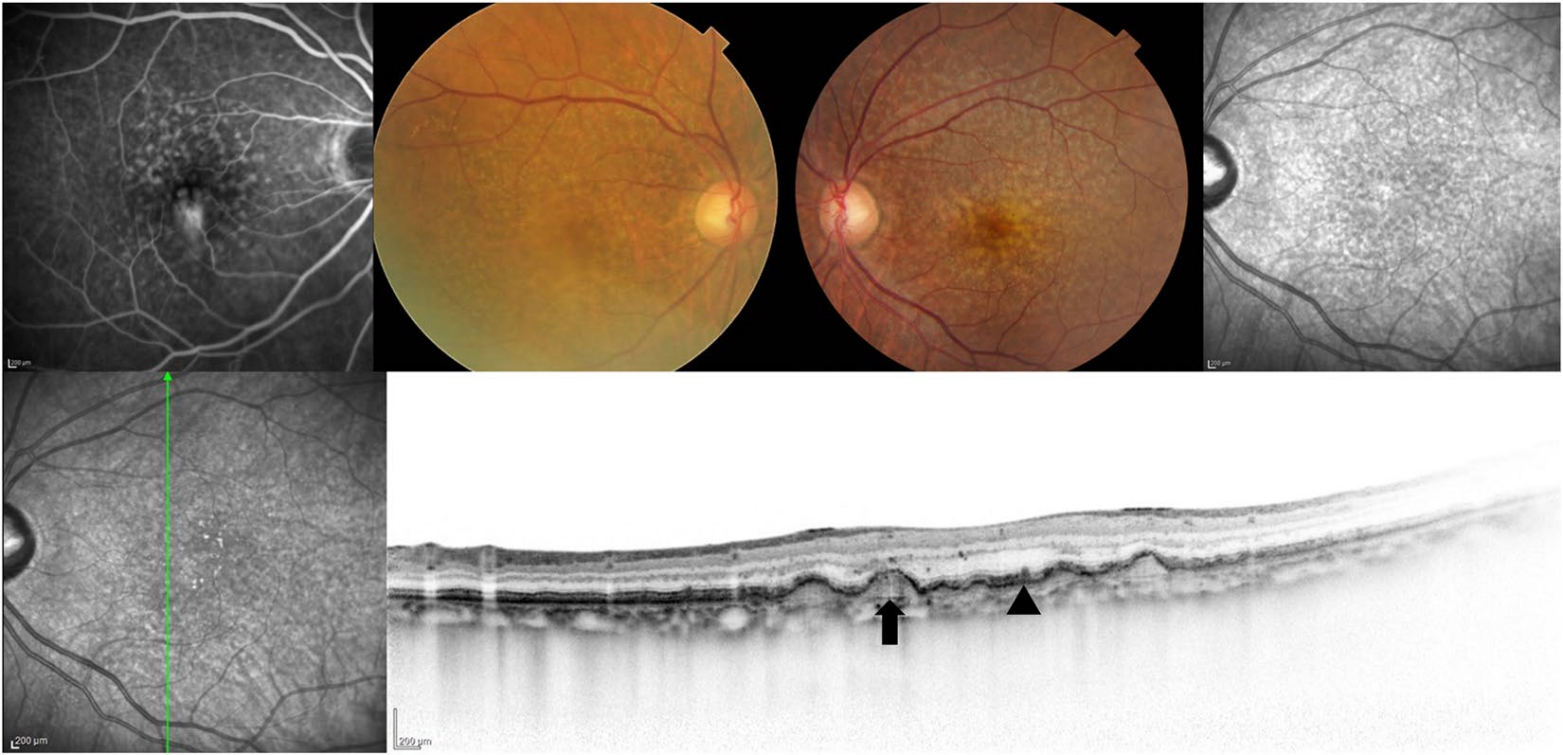
68-year-old male with unilateral retinal angiomatous proliferation and fellow eye with reticular pseudodrusen. (**A**,**B**) A color fundus photograph showed numerous pseudodrusen and soft drusen in the macula area of the right eye. Fluorescein angiography demonstrated hyperfluorescence corresponding to retina-retina anastomosis in the parafovea in the right eye. (**C**,**D**) A color fundus photograph showed soft drusen in the macula and pseudodrusen superior to the macula in the left eye. Near infrared reflectance revealed numerous areas of hyporeflectance corresponding to pseudodrusen in the left eye. (**E**,**F**) A vertical scan of optical coherence tomography demonstrated soft drusen (black arrow) and pseudodrusen (black arrowhead).

All patients underwent comprehensive ophthalmic examination in both eyes including best-corrected visual acuity (BCVA) assessment using the Landolt chart, slit-lamp biomicroscopy with or without a 78D lens intraocular pressure measurement, color fundus photography covering the posterior retina within 45 degrees, fluorescein and indocyanine green angiography (FA/ICGA), near-infrared reflectance (NIR), fundus autofluorescence (FAF) (HRA-2; Heidelberg Engineering, Dossenheim, Germany), and spectral-domain optical coherence tomography (SD-OCT) (Spectralis version 5.4 HRA + OCT). The subjects were classified into 4 groups according to the type of drusen seen in the fellow eye. Group 0: no drusen within 45 degrees in the posterior retina. Group 1: pachydrusen. Group 2: soft drusen. Group 3: pseudodrusen with or without soft drusen. Late phase ICGA was employed to differentiate pachydrusen, which exhibits punctate hyperfluorescent spots^23^, from soft drusen showing hypofluorescence^22^ (Figs 3 and 4). Late phase ICGA images were defined as ICGA images 10 minutes after dye injection. Eyes were diagnosed to have pseudodrusen if they showed a characteristic reticular pattern in at least one imaging modality including color fundus photography, FAF and NIR or subretinal drusenoid deposits with SD-OCT. (Fig. 5) The classification was confirmed by two independent graders (Y. F. and Y. S.) who was masked to the diagnosis of the affected eye. If their assessment differed, the final judgment was made by the third grader (H. I.). Since three patients were judged to have both soft drusen and pachydrusen in the fellow eye, they were excluded in this study. There were no patients who had both pseudodrusen and pachydrusen in the fellow eye.

The subfoveal choroidal thickness in the fellow eyes was measured as the vertical distance between the outer border of the retinal pigment epithelium and the choroidoscleral border, using SD-OCT images. A peripheral blood sample was collected before FA/ICG. Genomic DNA was purified using PUREGENE DNA Isolation Kit (Gentra Systems, Minneapolis, USA). Genotyping of *ARMS2* A69S (rs10490924) and *CFH* I62V (rs800292) was performed using Taqman genotyping assays with the 7300/7500 Real-Time PCR System (Applied Biosystems, Foster City, USA), as previously described^34^. Written informed consent was obtained from all participants.

### Statistical analysis

Statistical analyses were performed using Dr. SPSS for Windows (SPSS Inc, Tokyo, Japan). Differences in categorical variables were evaluated using chi-squared test. Differences in continuous variables between 2 groups or among 3 or 4 groups were evaluated by Mann-Whitney U test and analysis of variance, respectively. Log-rank test was performed to compare the cumulative incidence of fellow-eye involvement between the 2 groups. A P-value less than 0.05 was statistically significant.
